# Positive and Negative Correlates of Psychological Well-Being and Distress in College Students’ Mental Health: A Correlational Study

**DOI:** 10.3390/healthcare12111085

**Published:** 2024-05-25

**Authors:** Maria José Carvalho Nogueira, Carlos Alberto Sequeira

**Affiliations:** 1 School of Nursing of São João de Deus, Évora University, Largo do Sr. da Pobreza 2B, 7000-811 Évora, Portugal; 2 Comprehensive Health Research Centre (CHRC), Évora University, 7000-811 Évora, Portugal; 3 Center for Research in Health Technologies and Services: Health Research Network, From The Lab to The Community (CINTESIS@RISE), 4200-072 Porto, Portugal; carlossequeira@esenf.pt; 4 Nursing School of Porto (ESEP), R. Dr. António Bernardino de Almeida 830 844, 856, 4200-072 Porto, Portugal

**Keywords:** students, mental health promotion, nursing, psychological well-being, psychological distress

## Abstract

Background: Recognizing the positive or negative effects of students’ mental health promotes personal development, well-being, and academic success. Academic life exposes college students to multiple adjustments, demands, and vulnerabilities that can cause stress and mental health problems. This study aims to identify psychological well-being and psychological distress effects on college students’ mental health. Methods: A correlational study was designed involving a non-probabilistic 560 sample of students (446 women) aged 18 to 41 years (M = 19.6; SD = 1.68). An online self-report questionnaire was used including demographic, relational, academic, and health behaviors variables, and the measures: Mental Health Inventory; Satisfaction with Social Support Scale; Academic Life Satisfaction Scale; and Psychological Vulnerability Scale. Results: Regression analyses indicate that male students, dating, good academic performance, exercise, sleeping seven hours, satisfaction with social support, and academic life satisfaction were significant correlates (*p* < 0.05) of psychological well-being, with the last two having great weight in the model. Females, low income, aged 21–24 years, sleeping less than 6 h, moderate psychological vulnerability, and perception of vulnerability were significant (*p* < 0.05) negative effects of psychological distress. Conclusions: This study addresses the positive and negative effects of psychological well-being and distress in college students. Specific mental health promotion and morbidity prevention programs can improve students’ mental health literacy and resilience.

## 1. Introduction

The prevalence of distress in college students is higher than in the general population [1], and students’ mental health on campuses has been deteriorating. Students must succeed in all aspects of their lives, including academic life, and the literature review shows that mental health problems are common among college students [2]. The Healthy Minds Study—Data Report of 2018–2019 found that overall, 36% of students had depression, including significant and moderate depression; 31% had an anxiety disorder; 1% had screened positive for an eating disorder; 14% had suicidal ideation (previous year); and 24% have taken psychiatric medication (previous year) [3]. Recent evidence shows that a college student’s mental health is an intrinsic and essential asset to the student’s personal and full potential development and academic success [4,5]. Some authors argue that mental health issues are related to students’ academic failure and poor global functioning [6,7], while well-being is a strong indicator of physical, emotional, cognitive, academic, and interpersonal well-functioning [5,8,9], and low levels of well-being can lead to severe imbalances and vulnerabilities, like emotional instability, including suicide [10].

Additionally, the evidence points out some variables or correlates that have significant positive effects on psychological well-being and mental health, namely healthy lifestyle habits and good quality of sleep [2,11], academic life satisfaction (ALS) [12], and satisfaction with social support [13,14]. Similarly, the sense of protection and care received from family, friends, and the community increases self-esteem, optimism, and positive mood, reducing stress and feelings of loneliness and failure [6,15,16]. By contrast, the perception of the absence of social support is a well-known negative determinant of mental health problems [13] and is associated with higher levels of stress and depression [15,17]. Also in this sense, negative life events (NLEs), defined as early life adverse conditions or experiences [18], are vulnerabilities that may weaken students’ mental health [19,20]. Early adverse experiences can have long-lasting negative effects on peoples’ responses to stress, brain structures, and emotional processes [21]. Adverse life events are associated with memory and learning deficits, depression [22], suicidal behavior, and hopelessness [23].

Another variable indicated as a negative predictor of depression is psychological vulnerability (PV) or cognitive vulnerability [24]. PV is a negative cognition or a default cognitive and emotional schema that reflects harmful beliefs and dysfunctional attributes. These attributes make individuals more vulnerable to themselves in interaction with others, in the stress reaction, and in the ability to achieve goals. PV reflects dependence on external sources of approval [24]. Furthermore, the subjective awareness of feeling vulnerable, or the perception of vulnerability (PeV), globally affects the person’s functioning [25]. From a mental health perspective, the concept of vulnerability has evolved from an initial epidemiological approach to a more comprehensive conception (used by nursing), which includes the notion of unique perception and experience of feeling vulnerable or not [25,26]. The concept of vulnerability contains the uniqueness of the conditions in which each person finds themselves [24] or “a person’s experience of being unprotected and open to damage in threatening environments” (p. 337).

The literature review shows that research about students’ mental health focuses mainly on negative aspects [27,28] and is scarce in addressing students’ significant positive and negative effects as holistic persons. Furthermore, to the best of our knowledge, no study investigates predictive relations between students’ mental health and psychosocial and psycho-affective variables (cognitive vulnerability and perception, psychological well-being, psychological distress, negative life events, psychological vulnerability, and the perception of vulnerability).

The purpose of this study was to explore the existence of positive and negative effects on psychological well-being and distress in college students. In this sense, correlational designs are the most appropriate to inform about these influence effects. This knowledge can contribute to inform the development of appropriate programs to promote students’ mental health and well-being [5,8]. So, this study aimed to identify psychological well-being and psychological distress effects on mental health in college students.

## 2. Materials and Methods

### 2.1. Study Design and Participants

A cross-sectional correlational study design was used involving a non-probabilistic sample of 560 college students. All twenty-nine higher education institutions in the Lisbon District participated in this study, and the protocol was a web-based self-report survey. The sample size was calculated a priori using G × Power 3.1.9.7 software to conduct power analyses and determine sample sizes of statistical tests (*t*-tests, ANOVA, and regression analysis) [29]. In addition, an invitation was sent by email to all undergraduates to participate in the study. The email provided information about the aims and procedures of the study, the ethical aspects, and a link to access the survey covering all the variables and measures. The inclusion criteria were being more than eighteen years old and attending any course. Once completed, students had to validate the questionnaire before submitting it.

### 2.2. Ethical Approval and Consent to Participate

All methods of the study were carried out following relevant guidelines and regulations. The study was approved by the Ethics Committee Review Board (CE-ESEL-Flow 2017-208) and the Board Directors of all the institutions involved. All participants were informed about the purposes and implications of the study and gave written informed consent. They were also told that they could withdraw from the study at any time without penalty. Participants’ privacy was also assured. Data were encrypted and processed confidentially during the process. To ensure anonymity and confidentiality, a unique identification code for participants was used along with electronic filters to limit the data access to only the researcher and authorized personnel. At the end of the questionnaire, useful contacts in case of need or suffering were provided (student counseling center and other relevant resources and information). 

### 2.3. Measures and Instruments 

The variables and measures were selected based on the recent evidence that shows that psychosocial and affective variables have an important influence on college students’ mental health, as positive or negative determinants [22,23,28]. All authors gave their permission to use the instruments in the study. To collect demographic information, four items were used (gender, age, relationship/dating, and income level—Graffar Classification); for academic information, three items were included (course area categorized a posteriori in “Health” and “Other”, overall academic performance (self-reported), and type of education); and regarding participants’ health behaviors, two items were included (exercise and hours of sleep). 

The **Mental Health Inventory (MHI-38)** [30] measures mental health and comprises 38 self-report items distributed by two dimensions: psychological distress (PD) (3 subscales—22 items: anxiety, depression, and loss of emotional control) and psychological well-being (PW) (2 subscales—16 items: positive affect [11 items] and affective ties [3 items]). MHI-38 rates on a 6-point Likert-type scale as follows: 1 = all the time/always to 6 = none of the times. The MHI-38 score ranges from 38 to 228, comprising psychological distress (22 to 132) and psychological well-being (16 to 96). Higher scores on the MHI-38 correspond to better mental health except for the PD scale, which inverts the scores on its items. The reliability of MHI-38 in the present study was excellent (Cronbach’s *alpha* = 0.92).

The **Social Support Satisfaction Scale (SSSS)** [31] total score assesses the sense of protection and care received from family, friends, and the community. It has 15 self-report items and 4 subscales: satisfaction with friendship, intimacy, family satisfaction, and social activities. The scale is recorded on a 5-point Likert-type scale as follows: 1 = strongly disagree; 2 = strongly disagree; 3 = not agree or disagree; 4 = largely agree; and 5 = strongly agree. The reliability of the SSSS in the present study was very good (Cronbach’s *alpha* = 0.85).

The **Academic Life Satisfaction Scale (ALSS)** [12] measures the student’s academic life satisfaction. The ALSS is a self-completed scale with eight items and two dimensions: personal satisfaction (four items: students’ abilities, perception of academic performance, and relationships with colleagues and teachers) and satisfaction with the academic environment (four items: physical and pedagogical environment, commitment to the course, extracurricular activities on campus, and study conditions). ALSS scores are determined on a 5-point Likert-type scale as follows: 1 = strongly disagree; 2 = strongly disagree; 3 = neither agree nor disagree; 4 = agree; 5 = strongly agree. The total score is the sum of all items (ranges between 8 and 40), and higher scores reflect better academic life satisfaction. The reliability of ALSS in the present study was very good (Cronbach’s *alpha* = 0.80). 

The **Negative Life Events Inventory (NLEI)** [18] assesses negative life events (frequency, impact, and severity) experienced by students. The NLEI has 25 items distributed by 4 subscales: dysfunctional family environment (family conflicts, separations, and substance abuse by caretakers); psychological abuse (being humiliated, rejection, emotional coldness, disproportionate punishment/expectations, and verbal or written threats); separation and loss (recurrent, prolonged, and definitive separations); physical or sexual abuse (physical abuse, aggression, forced viewing of sexual intercourse, sexual stimulation, and forced sexual intercourse). The NLEI is scored as a *Global Index of Presence* by counting the frequency on a 5-point Likert-type scale (0 = never; 4 = many times) and in terms of the *Severity Index* by scoring the negative impact on a 5-point Likert-type scale as follows: 1 = no impact/consequence; 5 = extremely negative. In the present study, only the *Global Index of Presence* and *Severity Index* were used. The reliability of the NLEI in the present study was very good (*Cronbach’s alpha* = 0.83). 

The **Psychological Vulnerability Scale (PVS)** [32] measures psychological vulnerability, reflecting maladaptive cognitive pattern schemas and harmful beliefs, such as dependence, perfectionism, need for external sources of approval, and generalized dysfunctional attributions. The PVS is a six-item self-administered instrument, e.g., “*I need approval from others to feel good about myself*”, that rates on a 5-point Likert-type scale as follows: 1 = do not describe me at all to 5 = describes me very well. The total score is the sum of all items (varying between 6 and 30). Thus, higher scores indicate greater psychological vulnerability. The reliability of the PVS in the present study was acceptable (Cronbach’s *alpha* = 0.73). 

A single question about the ***Perception of Vulnerability*** (PeV), namely “*do you feel vulnerable from the point of view of your mental health?*”, assesses the state of mind related to mental health, with the following rating on a 5-point Likert-type scale: 1 = not vulnerable to 5 = extremely vulnerable. To fully capture the complexity of vulnerability, we combined this question with the above measures for a more comprehensive assessment. Effectiveness was achieved by comparing the results to established measures like psychological vulnerability.

### 2.4. Statistical Analysis

The SPSS 25.0 (IBM Inc., Armonk, NY, USA) statistical package for Windows was used for data analysis, including descriptive and inferential statistical analysis. Reliability was assessed using Cronbach’s alpha coefficient (values of α > 0.7 were assumed acceptable for internal consistency). Statistical significance was assumed at the confidence level of *p* < 0.05.

A multiple hierarchical linear regression (MHLR) analysis was used to identify the significant effects on mental health. The models’ assumptions were generally satisfied: the linearity of the relationship between the independent variables and the dependent variable (graphical analysis); the independence of residuals (Durbin–Watson test); the normality of residuals (Kolmogorov–Smirnov test); multicollinearity (no multicollinearity if VIF < 10 and tolerance > 0.1); and the homogeneity of variances (graphical analysis).

Student’s *t*-test was used when comparing two groups, and one-way ANOVA was used when comparing three or more means. The homogeneity of variances was verified using Levene’s test, with *p* values greater than 0.05. When ANOVAs were used to compare multiple means, and the results showed significant differences, post hoc tests were used to highlight which groups differed from each other. Bonferroni was used whenever up to five comparisons were carried out (Marôco, 2011). When the conditions for applying the *t*-tests and ANOVAs were not met, and important assumptions had been violated, although the sample was greater than 500 participants, non-parametric tests were used. The Mann–Whitney U was used when there were two groups to compare. To test whether two qualitative variables were associated (categories), the chi-square test was used. When the application conditions were not met, the Fisher test or Monte Carlo simulation was used as an alternative. When the *p* result of the chi-square/Fisher test was higher than the significance level (*p* > 0.05), it was considered that there was no association between variables. To identify which categories were associated with each other, the adjusted residual value (RAjust > 1.5 = associated categories) was used [29]. In addition, nominal variables were converted into dummy variables [29], i.e., assigning numerical values to categorical variables. When dealing with a binary variable (only two categories), we chose the category to be the reference group represented by zero (0), while the other category was represented by one (1) in the dummy variable. For variables with more than two categories, we added sequential numbers (3)..(4). 

Variables with significant correlations in previous bivariate analyses with MHI-38 (total scale) were included in the RLMH models (MHI-38’s PD and PW subscales) sequentially by blocks or logical groups of independent variables, estimating in stages the degree of explanation of the explained variance of the variables of the two models. Independent variables were entered in following the order: sociodemographic variables (gender, income level, relationship/dating, and age group), academic variables (course area, self-classification of academic performance, and type of education), health behaviors (exercise physical/sport, average hours of sleep per day), and psycho-affective factors (satisfaction with social support—total score, psychological vulnerability—total score, academic life satisfaction—total score, perception of vulnerability, and NLEI—Severity Index). The variables age group and course area, due to theoretical affinities that were desired to be studied, were included in the models, although they had not previously been shown to be significant.

For data cleaning, we removed duplicate entries in the dataset, and for handling missing values and outliers, we transformed/imputed estimated values (mean, median, and mode) [29].

## 3. Results

### 3.1. Demographic and Health Characteristics

Participants were mostly female (79.6%), with an age mean of 19.6 years (SD = 1.7), having relationships or dating (43%). More than half attended the 2nd year and public institutions (82.7%), and almost all (97.3%) perceived their academic performance as positive. Overall, 52.5% of participants exercised, most reported sleeping only 6 or 7 h per night, and most felt that this was not enough time. Notably, 37.8% of the participants fell under the low-income level. Table 1 summarizes the participants’ characteristics. 

The results from the MHI-38 reveal very good mental health levels (total scale [38; 228]: M = 158.87; SD = 29.49) in the participants. An identical result was found in the positive dimension (PW subscale [14; 84]: M = 52.46; SD = 11.70) and a moderate level of emotional suffering in the negative dimension (psychological distress subscale [24; 144]: M = 107.41; SD = 19.42). The results from the Social Support Satisfaction Scale also disclosed high levels (the total SSSS [15; 75]: M = 52.3; SD = 10.29) of satisfaction with social support. The SSSS subscale—satisfaction with friends (SF [5; 25]: M = 18.70; SD = 3.89) had the highest value, and the subscale social activities (SA [3; 15]: M = 7.90; SD = 2.98) had the lowest. 

Overall, participants were satisfied with their academic life (the total ALSS [8; 40]: M = 29.2; SD = 5.3), and we found identical scores in the subscales. However, personal satisfaction (SP [4; 20]: M = 14.61; SD = 2.92) scored slightly higher than satisfaction with the academic environment (SAE [4; 20]: M = 14.59; SD = 3.19). The results from the Negative Life Events Inventory showed a low frequency (Presence Index [0; 25]: M = 7.27; SD = 7.24) and low negative impacts of negative life events (Severity Index [0; 20]: M = 4.19; SD = 2.19) in the sample.

Yet, for those who reported negative life events (Global Index [0; 4] M = 1.41; SD = 1.34), the negative impact experienced (M = 4.19) was significant. The results indicate that participants had a moderate psychological vulnerability (PVS [5; 30]: M = 16.6; SD = 5.3). The results from the single question about the perception of vulnerability revealed that students did not perceive themselves as vulnerable (PeV [1; 5]: M = 2.04; SD = 0.96).

### 3.2. Psychological Well-Being

To find the possible factors associated with psychological well-being (PW) a hierarchical multiple linear regression (HMLR) was performed. The PW final model involved the total score of the PW subscale, the total scores of SSS and ALS measures, and the variables included in Table 2. Variables entered in the model were demographic, academic, relational, health behavior, and psycho-affective variables (cognition and perception) as independent variables, which explained 53.8% of the total variance of the psychological well-being model. Academic variables were related to the lowest value (5.6%) of the model, followed by demographic (9.2%) and health behaviors (9.4%), explaining similar values. The psycho-affective variables explained the highest variance value (29.6%) of the psychological well-being model. The results showed that women had significantly lower psychological well-being values than men (*β* = −3.55, *p* = 0.024) and a lower economic level (*β* = −1.78, *p* = 0.025).

Participants with satisfactory relationships or dating had the highest psychological well-being (*β* = 3.44, *p* = 0.012), while the oldest age group (21 to 24 years old) exhibited lower psychological well-being (*β* = −4.91, *p* = 0.002). Students with better self-rating/best self-classification in academic performance (*β* = 3.48, *p* = 0.001), as well as those who did not perform physical exercise (*β* = 3.15, *p* = 0.016), slept seven or more hours per night (*β* = 4.08, *p* = 0.002), and were most satisfied with social support (*β* = 0.42, *p* = 0.001) and their academic life (*β* = 0.33, *p* = 0.005), reported having better psychological well-being. By contrast, those with a higher perception of vulnerability (*β* = −1.85, *p* = 0.006) indicated worse psychological well-being. Satisfaction with social support and academic performance had the most significant correlations with psychological well-being. 

### 3.3. Psychological Distress 

Table 3 displays the association with psychological distress (PD). The model involved the total score of the PD subscale; the total scores of the SS, PV, PeV, ALS, and NLEI—Severity Index measures; and the variables included in Table 3. The variables entered in the model were demographic, academic, relational, health behavior, and psycho-affective variables as independent variables. The hierarchical multiple linear regression (HMLR) final PD model analysis showed that demographic, relational, academic, health behavior, and psycho-affective variables as independent variables explained 55.4% of the psychological distress total variance. Academic variables explained the lowest value (1.4%) of the variance, demographic variables explained 5.1%, and health behaviors explained 13.2%. 

The psycho-affective variables explained the largest share of variance (R^2^ = 35.7%) in the total model, as shown in Table 3. The results also showed that women had significantly higher psychological distress values than men (*β* = −6.79, *p* = 0.013), as well as the oldest students (21 to 24 years; *β* = −4.91, *p* = 0.002). Those who slept 7 or more hours (*β* = −6.37, *p* = 0.006) and were most satisfied with social support (*β* = −0.52, *p* = 0.001) and academic life (*β* = −0.55, *p* = 0.005) had lower values of PD. 

Those with greater psychological vulnerability (*β* = 0.91, *p* = 0.001) and those with a higher-level perception of vulnerability (*β* = 5.27, *p* = 0.001) obtained higher psychological distress values. The most significant variables in the regression psychological distress model were the perception of vulnerability, satisfaction with social support, and psychological vulnerability. 

## 4. Discussion

This study aimed to identify positive and negative college students’ mental health variables. Based on RLMH models obtained for psychological well-being and psychological distress, the results highlighted relevant contributions of the psycho-affective variables in explaining both models. 

**The psychological well-being model** significantly explained a relevant percentage value (53.8%) of the total variance, which is relevant in social sciences, highlighting the positive effect of the variables (demographic, relational, academic, health, and psycho-affective) as independent variables. The RLMH analyses indicated that being male, having a satisfactory relationship, good academic performance, exercising, sleeping seven hours per night, having satisfaction with social support, and academic life satisfaction were students’ significant positive effects on psychological well-being. Unsurprisingly, being female had a negative effect on psychological well-being [33] since prior studies showed that it is common for women to report significantly lower psychological well-being values than men [5,8,34]. Recently, these differences in psychological well-being have mainly been associated with cultural aspects, discrimination, or gender violence that cumulatively lead to more significant stress and psychological strain on women [35,36]. Also, satisfactory dating was a positive predictor of psychological well-being, tied to establishing good intimate relationships, a positive developmental marker for young adults [37,38] with a positive influence on students’ mental health. The model showed a negative correlation between older students and psychological well-being. However, some studies found that well-being gradually increases between 18 and 25 years [39]. 

Therefore, future studies are needed to clarify and add robustness to the results. This effect might be due to the positive influence on the student’s maturity and consolidation of professional plans [39] but can also be explained by the negative impact of financial pressure, role changes in life, interpersonal relationships, and students’ market perspectives of employment [40]. However, the mediating role of these associations with psychological well-being was not studied. Thus, further studies are needed to clarify these relationships. As expected, a high-income level was a positive predictor of psychological well-being primarily due to the positive effect of financial security and comfort [8].

Positive academic performance perceptions were documented to be a very significant predictor of the psychological well-being model (5.6% of explaining variance), and this result is consistent with previous findings; for instance, having “good grades” is one of the most significant effects on students’ psychological well-being [41,42]. As expected, sleeping more than seven hours and having regular exercise were robust protective effects, as they significantly contributed to the psychological well-being model. In addition, evidence consistently reports significant positive associations between students’ physical activity patterns and sleep hygiene with a better level of mental health [43] and students’ global functioning [44]. Endorphins produced during exercise improve cognition and memory [45], improve self-esteem, and decrease the adverse effects of stress and mental and physical tension [46]. Predictably, the results showed a relevant sleep weight in explaining the psychological distress model (13.2% of variance). Those who slept less than six hours per night had higher values of psychological distress, confirming previous studies reporting positive correlations between poor sleep hygiene and high levels of distress [7,47]. In addition, recent studies show that sleep disturbances and deprivation predict distress and are associated with psychological symptoms and depression [28]. Moreover, students’ sleep quality worsens during high-stress periods [7,11], and sleep quality and mental health levels improve with regular physical exercise [2,43]. 

This knowledge is noteworthy and particularly relevant for health professionals who can use it to diagnose sleep-related disorders and poor sleep quality in order to implement accurate educational programs. Such programs can empower students to adopt healthier sleep hygiene patterns and behaviors on campus [11,44,47,48]. 

Together, satisfaction with social support and satisfaction with academic life were two robust positive variables since both explained 29.6% of the psychological well-being model. These results are generally in line with prior findings showing that students more satisfied with social support, parental involvement, and their academic life obtained higher values of well-being [16,49,50,51]. The beneficial effect of the perception of social support is provided mainly by family and friends. A positive perception of social support fosters confidence, positive self-esteem, self-acceptance and identity, participation, and sharing [6,15]. These positive feelings, in turn, help to face adversities since they release stress and manage anxiogenic situations and physical and psychic tensions [13]. Therefore, social and cultural campus initiatives are needed to encourage students’ involvement in enjoyable relationships and extracurricular social activities with peers. Young adults’ interaction with peers is an essential growth dimension, as they feed positive emotional states and self-acceptance [15,51]. Previous findings also highlight that experiences of satisfaction with academic life have a protective effect on students’ academic success and adaptation on campus [12,38]. Therefore, students’ satisfaction with academic life on campus must be promoted, as well as students’ interpersonal skills, ultimately increasing students’ harmonious functioning, decision-making, and academic success. 

The psychological distress model showed a solid proportion of the total variance (55.4%) of psychological distress, explained by demographics, health behaviors, and psycho-affective as independent variables. Unsurprisingly, women had significantly higher psychological distress values than men and a substantial weight in the model. This result is similar to previous findings that have explained that this is due to cumulative variables like academic and adjustment demands and several gender-related challenges [33,36]. The psycho-affective variables explained the highest value of the psychological distress model (35.7% of the variance). Expectedly, students with higher values of psychological vulnerability and higher perceptions of vulnerability obtained higher values of psychological distress since psychological vulnerability is related to negative schemas that reflect harmful beliefs, dysfunctional attributes, and interpersonal dependence. These results are similar to recent studies that found a significant positive relationship between psychological distress, psychological vulnerability, and depression [24,52,53]. 

Additionally, the perception of vulnerability or the *feeling of being vulnerable* was a stronger correlate of the psychological distress model, aligned with Rogers’s theoretical model (Rogers, 1997 [25]). Rogers argues that feeling vulnerable produces distress and affects the person’s functioning globally. The perception of vulnerability was significantly correlated with psychological vulnerability and psychological distress, indicating that students can self-assess their degree of vulnerability, and this is a bidirectional relationship.

This result reveals a promising indicator for the early screening of self-perception of vulnerability in students. Therefore, asking a question such as *do you feel vulnerable from a mental health point of view?* can appraise the state of mind. This is a simple instrument that may help health professionals and teachers quickly screen students at risk or needing professional help. Despite the negative effect of childhood adversity on depression and insomnia symptoms among students [19,20,22], hopelessness, and suicidal behavior [23], no significant contributions to negative life events were found in the psychological distress model. These findings improve the understanding of the specific effects of several variables on student’s psychological well-being and distress. Our results can inform future investigations and mental health nursing interventions. 

Specific data help health professionals and universities be ahead of students’ needs before students experience the predictable stress of college life. The study results highlight the need to support and strengthen students’ social and academic networks to reduce anxiety and psychological distress. Intervention programs to prevent mental suffering on campus can involve the *early detection* of mental health problems, providing *first aid in mental health* on campus, providing pedagogical support, offering *soft skills workshops* and a credit-based course (communication skills, stress management, aggressive behavior, self-knowledge, relaxation exercises, and mindfulness), and supporting students’ social and academic networks.

The study’s strengths are associated with finding robust effects of psychological well-being and distress in college students. Also, some limitations must be highlighted to interpret the results. Firstly, the risk of response bias associated with self-report measures and social desirability can lead participants to over- or under-report certain types of behavior, depending on whether they consider it socially acceptable. As a result, self-report measures may lower the internal validity of the data. Thus, future studies can profit from using different techniques, such as peer review, strengthening the internal validity. Secondly, correlational and cross-sectional designs cannot establish causality definitively. Also, care should be taken when generalizing the results to the entire population and rural areas since only students from the largest urban area in Portugal (Lisbon District) participated in the study, as well as due to the non-probabilistic sample. Finally, considering gender-specific risk, the sample ratio of men vs. women may present a particular bias, limiting the generalization. However, the sensitivity analysis performed (effect size calculations and pattern analysis) on baseline analysis vs. sensitivity scenario for the two gender groups showed that the findings remain consistent across scenarios (age, socioeconomic status, and geographic location), suggesting robustness.

Despite the very good mental health levels exhibited in the sample, there may still be subgroups within the student population that are at risk. It is crucial to identify these subgroups and provide targeted interventions to ensure that no students are left behind. Additionally, maintaining and possibly enhancing the current support systems that contribute to these positive mental health outcomes is important to sustain these levels over time. Continuous monitoring and assessment are also necessary to promptly address any emerging issues.

Future research must use a longitudinal design and structural equation modeling to better understand the dynamics between variables over time, samples from wider geographic areas, and students from all grades and areas of study. These strategies can contribute to generating more robust and conclusive evidence that can be generalized to broader contexts.

## 5. Conclusions

This study adds specific data about protective and vulnerability factors based on two significant models explaining students’ psychological well-being and psychological distress. Positive influences on psychological well-being included being male, having an intimate relationship, good academic performance, regular exercise, sleeping seven hours per night, and satisfaction with social support and academic life. Conversely, being female, having a low income, being aged between 21 and 24 years, sleeping less than six hours, having moderate psychological vulnerability, and having a high perception of vulnerability were negative effects on psychological distress. These results can help professionals implement evidence-based programs to promote mental health literacy, well-being, and resilience on campus, open to all students.

These findings can be used to develop tailored student intervention strategies to increase mental health and psychological support and prevent or minimize their emotional suffering. This intervention process must be a health priority considering that poor mental health limits the student’s development potential, both academically and personally.

## Figures and Tables

**Table 1:** Demographic, academic, and health behavior characteristics of the sample.

Variables	N°	Percentage	M (SD)
Age			19.60 (1.68)
18–20	428	76.4	
21–24	132	23.6	
Sex			
Women	446	79.6	
Mem	114	20.4	
Relationship/dating			
Yes	294	43.0	
No	266	47.5	
Other	53	9.4	
Income level *			
High	164	29.3	
Medium	184	32.9	
Low	212	37.8	
Course **			
Health	343	61.3	
Other	217	38.8	
Type of Education			
Public	416	74.2	
Private	120	20.3	
Military/Police	24	3.5	
Academic performance ***			
Mediocre ^(b)^	15	2.7	
Sufficient	123	22.0	
Good	341	60.9	
Very good	74	13.20	
Great	7	1.30	
Physical Exercise ^¥^			
No	266	47.5	
Yes	294	52.5	
Daily	59	20.1	
2 to 3 times per week	136	46.3	
Once a week	83	28.2	
Sleeping hours ^(a) ¥^			
≥8 h	33	5.9	
7 to 8	205	36.6	
6 to 7	230	41.1	
≤5 to 6	92	16.4	

* Graffar Classification renewed into three levels; ** categorized a posteriori by the authors; *** overall academic performance (self-reported); ^(a)^ average hours/per night; ^¥^ during school; ^(b)^ minimum value to be successful in the curricular unit.

**Table 2:** Hierarchical multiple linear regression of psychological well-being model.

Stage	Variables	Δ R^2^	*β*	Std. Error
Step 1	Demographic and Relational	0.092 ***		
	Gender		−3.55 *	1.57
	Income level		−1.78 *	0.79
	Satisfactory relationship/dating		3.44 *	1.36
	Age group		−4.91 **	1.57
Step 2	Academics	0.056 ***		
	Health course		−1.40	1.46
	Academic performance ^(a)^		3.48 ***	0.92
Step 3	Health Behaviors	0.094 ***		
	Physical exercise/sport ^¥^		3.15 *	1.31
	Sleeping hours ^(b) ¥^		4.08 **	1.31
Step 4	Psycho-Affective	0.296 ***		
	Satisfaction with social support		0.42 ***	0.06
	Academic life satisfaction		0.33 **	0.12
	Total R^2^	0.538 ***		

^(a)^ Self-evaluation; ^(b)^ average hours/per night; ^¥^ during class period; * *p* ≤ 0.05 ** *p* ≤ 0.01 *** *p* ≤ 0.001; *β*—unstandardized coefficients.

**Table 3:** Multiple hierarchical linear regression of psychological distress model.

		Δ R^2^	*β*	Std. Error
Step 1	Demographic and Relational	0.051 ***		
	Gender		−6.79 *	2.70
	Income level		−2.13	1.36
	Intimate relationship/dating		0.59	2.34
	Age group		6.07 *	2.71
Step 2	Academics	0.014 ***		
	Year		0.51	2.39
	Health course		−0.41	2.58
	Academic performance		3.15	1.62
Step 3	Health Behaviors	0.132 ***		
	Physical exercise/sport		2.79	2.27
	Hours of sleep		6.37 **	2.29
Step 4	Psycho-Affective	0.357 ***		
	Satisfaction with social support		−0.52 ***	0.11
	Psychological vulnerability		0.91 ***	0.22
	Vulnerability perception		5.27 ***	1.12
	Academic life satisfaction		−0.55 **	0.19
	NLEI—Severity Index		0.02	0.47
	Total R^2^	0.554 ***		

* *p* ≤ 0.05; ** *p* ≤ 0.01; *** *p* ≤ 0.001; *β*—unstandardized coefficients; NLEI—Negative Life Events Inventory.

## Data Availability

The data supporting this study’s findings are available upon request from the corresponding author. However, the data are not publicly available due to privacy or ethical restrictions.
